# Machine Learning Protein Motif Fusion for Avid Sensing, Intracellular Reporters, and Crystalline Assemblies

**DOI:** 10.1063/4.0001109

**Published:** 2025-10-27

**Authors:** Ethan T Shields, Emma N Magna, Callie K Slaughter, Pegah Eizadkhah, Christopher D Snow

**Affiliations:** Colorado State University, Fort Collins, CO, USA

## Abstract

Modern machine learning protein design methods offer increased success rates for de novo protein binder design and building new protein structures that embed existing protein motifs. Some applications in biomolecular recognition and structural biology depend on the precise spatial arrangement of functional protein motifs. For example, we are using a pipeline consisting of RFDiffusion, ProteinMPNN, and AlphaFold2 to design novel proteins that can recognize specific ubiquitinated histone form, despite the considerable conformational flexibility of the conjugated ubiquitin. We are also using these methods to design new proteins for intracellular binding of target peptides. For example, we have used AlphaFold2 to predict which part of a viral protein serves as the epitope for an antibody, or ProteinMPNN to rescue otherwise inactive intracellular single-chain antibody fragments. Finally, we are also using the latest machine learning methods for protein structure prediction and design to engineer building blocks for high-precision biomaterials. In particular, we are designing and applying porous crystals made of protein building blocks, DNA building blocks, or both. The resulting “molecular pegboard” materials can capture and organize functional guest molecules with high precision, capacity, and stability. Ultimately, the resulting scaffold crystals are intended to provide a new route for the routine, high-throughput structure determination of guest biomolecules.

